# Supplementary material for: Splice-Junction-Based Mapping of Alternative Isoforms in the Human Proteome
Source: Cell Rep. Author manuscript; Available in PMC 2020 Jan 15. (PMC6961840; doi:10.1016/j.celrep.2019.11.026)
Supplement: 2 [file NIHMS1546469-supplement-2.zip › DF1/PXD010154/Thyroid/Thyroid_10_TG_SCGPTPCQLQSEQAFLR.pdf]

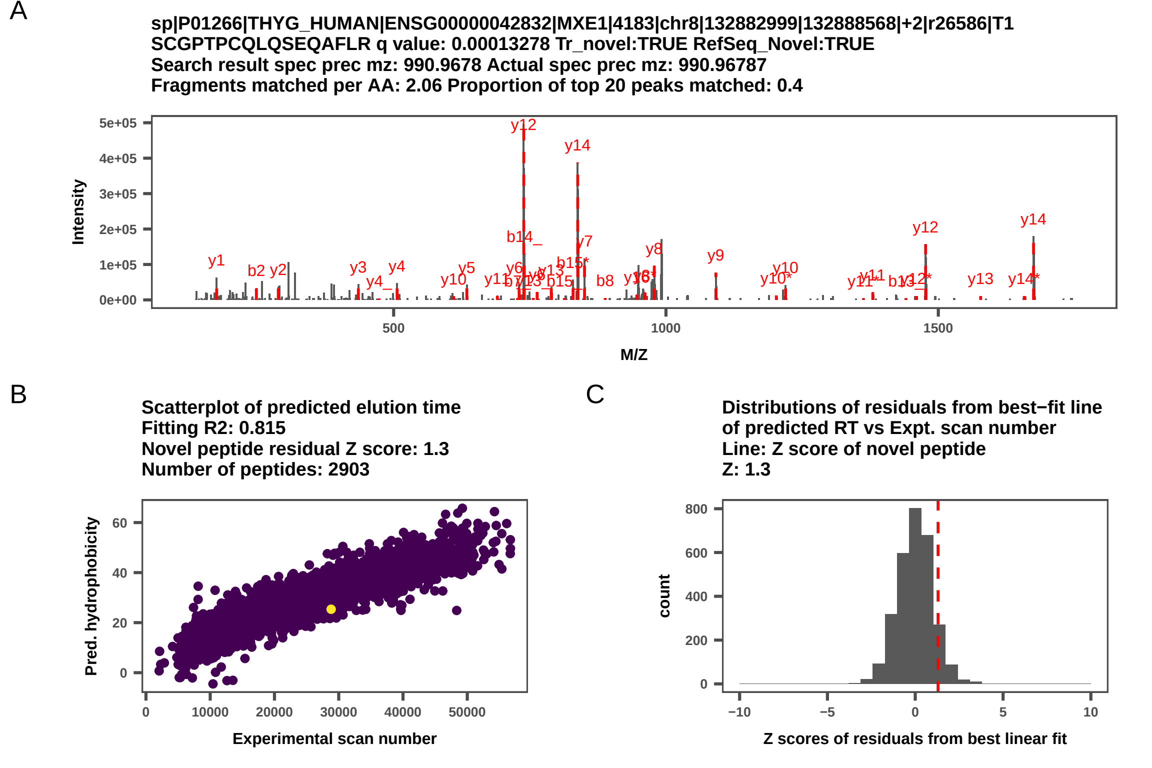
A
sp|P01266|THYG_HUMAN|ENSG00000042832|MXE1|4183|chr8|132882999|132888568|+2|r26586|T1
SCGPTPCQLQSEQAFLR q value: 0.00013278 Tr_novel:TRUE RefSeq_Novel:TRUE
Search result spec prec mz: 990.9678 Actual spec prec mz: 990.96787
Fragments matched per AA: 2.06 Proportion of top 20 peaks matched: 0.4
Intensity
5e+05
4e+05
3e+05
2e+05
1e+05
0e+00
500
1000
1500
M/Z
B
Scatterplot of predicted elution time
Fitting R2: 0.815
Novel peptide residual Z score: 1.3
Number of peptides: 2903
Pred. hydrophobicity
60
40
20
0
10000
20000
30000
40000
50000
Experimental scan number
C
Distributions of residuals from best−fit line
of predicted RT vs Expt. scan number
Line: Z score of novel peptide
Z: 1.3
count
800
600
400
200
0
−10
−5
0
5
10
Z scores of residuals from best linear fit
